# Macroporous Cell-Laden Gelatin/Hyaluronic Acid/Chondroitin Sulfate Cryogels for Engineered Tissue Constructs

**DOI:** 10.3390/gels8090590

**Published:** 2022-09-16

**Authors:** Gulshakhar Kudaibergen, Madina Zhunussova, Ellina A. Mun, Yerlan Ramankulov, Vyacheslav Ogay

**Affiliations:** 1 Stem Cell Laboratory, National Center for Biotechnology, Nur-Sultan 010000, Kazakhstan; 2 School of Science and Humanities, Nazarbayev University, Nur-Sultan 010000, Kazakhstan

**Keywords:** cryogels, gelatin, hyaluronic acid, chondroitin sulfate, mesenchymal stem cells

## Abstract

Cryogels are a unique macroporous material for tissue engineering. In this work, we study the effect of hyaluronic acid on the physicochemical properties of cryogel as well as on the proliferation of a 3D model of mesenchymal stem cells. The functional groups of the synthesized cryogels were identified using Fourier transform infrared spectroscopy. With an increase in the content of hyaluronic acid in the composition of the cryogel, an increase in porosity, gel content and swelling behavior was observed. As the hyaluronic acid content increased, the average pore size increased and more open pores were formed. Degradation studies have shown that all cryogels were resistant to PBS solution for 8 weeks. Cytotoxicity assays demonstrated no toxic effect on viability of rat adipose-derived mesenchymal stem cells (ADMSCs) cultured on cryogels. ADMSC spheroids were proliferated on scaffolds and showed the ability of the cryogels to orient cell differentiation into chondrogenic lineage even in the absence of inductive agents. Thus, our results demonstrate an effective resemblance to extracellular matrix structures specific to cartilage-like microenvironments by cryogels and their further perspective application as potential biomaterials.

## 1. Introduction

Scaffolds play an important role in tissue engineering as they are often used as a basis for cell proliferation. Such polymers must be biodegradable and biocompatible, have a 3D structure, mechanical strength, etc. [1]. To date, many methods are known to obtain scaffolds, however, most of them require specific equipment, high temperatures/pressures or use of hazardous organic solvents [2]. Compared to traditional methods, cryopolymerization is a simpler method for obtaining macroporous polymers. The process of cryopolymerization has been studied for a long time, and Professor Lozinsky V.I. made a huge contribution in this direction. Cryogels are three-dimensional macroporous materials [3] and are formed at a low solvent temperature ((sub) zero) [4]. At negative temperatures, most of the solvent freezes, and some remains unfrozen in the liquid microphase, where the dissolved substances are concentrated and enter into chemical reactions [5]. After polymerization is completed, the ice crystals are thawed at room temperature, thus melting the formed ice crystals and forming a porous cryogel with a pore size of 1 to 200 μm [6,7,8].

When comparing hydrogels and cryogels, hydrogels are crosslinked 3D networks with a high water content [9,10]. Hydrogels are known to swell better than cryogels, however; Hixon et al. synthesized silk fibrin cryogels and hydrogels and showed that cryogels can swell to a greater extent [11,12,13]. Cryogels, on the other hand, have such important properties as flexibility, large pores, high mechanical strength, short diffusion paths and good biocompatibility [14,15]. Cryogels can also have shape-memory properties and can be synthesized in various forms, such as powder, columns, beads, membranes, monoliths, and injectable forms [16,17,18,19,20,21]. The high porosity makes them suitable for use as macroporous cell-laden scaffolds for tissue constructions [13]. These cryogels can be used in a variety of biomedical applications, including diagnostics, therapeutics, drug delivery and tissue engineering.

Many cryogels were prepared from natural polymer-based materials and molecules because of their nontoxicity, biodegradability, biocompatibility and non-immunogenicity. Among others, glycosaminoglycans (GAG)-based cryogels are of a particular interest [22,23]. GAGs are a family of natural, negatively charged unbranched heteropolysaccharides composed of disaccharide repeating units that are present on cell surfaces, basement membranes and extracellular matrices (ECM) [24]. Due to their properties, natural polymers are often used in tissue engineering [25,26].

One of the most widely used natural polymer is gelatin (Gel)—a biopolymer derived from animal collagen, the major protein component of cartilage, but with relatively low antigenicity and a lower cost than collagen, which is widely used in clinical applications [18,19,27]. Gel does not have an ordered structure, unlike collagen, and is soluble in water; therefore, it is often used in the formation of cryogels as well as being modified from methacryloylated gelatin [28]. Hyaluronic acid (HA) or hyaluronan is a high-molecular-mass polysaccharide, also a natural polymer, a non-sulfated form of GAG and major component of ECM. HA regulates cell–cell adhesion, cell migration, growth and differentiation [29,30,31]. HA-based cryogels are biocompatible and biodegradable, and therefore these are often used as cell seeding scaffolds to repair cartilage defects [32].

Another good example of a natural polymer is chondroitin sulfate (CS). CS is a sulfated form of GAG, composed of a chain of alternating units of glucuronic acid and *N*-acetylgalactosamine, the latter being sulfated at O6 [33]. CS attaches to proteins as part of proteoglycan, an important component of cartilage, and its inclusion in a scaffold allows for the secretion of proteoglycans and type II collagen [34,35].

Mesenchymal stem cells (MSC) have found use in a wide range of fields, predominantly in regenerative medicine, e.g., bone and nerve regeneration [36,37,38]. This can be explained by high proliferative ability, self-renewal and differentiative potential, an abundance of their sources in various anatomic sites as well as the relative ease of isolation [39,40,41]. The main concern related to the capacity of MSCs to differentiate into various types of cell lineages spontaneously can be successfully overcome by utilizing specific physical, chemical and biological regulators to control cell fate [42]. It was shown that MSCs that grow in three-dimensional conditions have improved cell–cell and cell–matrix interactions compared to cells cultivated on monolayer, which results in elevated surveillance, proliferative abilities and stemness maintenance [43,44,45].

To date, similar works are known on the creation of crosslinked cryogels based on Gels, HA and CS using the crosslinking agent *N*-(3-dimethylaminopropyl)-*N*-ethylcarbodiimide hydrochloride (EDC) [2,46,47]. Kuo et.al. investigated macroporous gelatin/chondoitin-6-sulfate/hyaluronan (GCH) cryogel and GCH/chitosan cryogel for scaffold mechanical properties and chondrocytes response [48]. Later, the same group synthesized the highly elastic gelatin/chondroitinsulfate/hyaluronan/chitosan cryogel and determined that cryogel showed an enhanced chondrogenic phenotype from gene expression. They demonstrated that mechanical stimulation could promote the effectiveness of adipose-derived stem cell differentiation toward the chondrogenic lineage by co-culture chondrocytes with adipose-derived stem cells in vitro [47]. Other studies have synthesized scaffolds of gelatin/chondroitin sulfate/hyaluronan/polyvinyl alcohol (GCH-PVA) with orientated micro-tubule structure and good hydrophilicity, which were fabricated by unidirectional freeze-drying method mimicking the composition and structure of tracheal cartilage extracellular matrix. The incorporation of PVA improved gene expression and resulted in better proliferation of mouse bone marrow mesenchymal stem cells [49].The main difference between our work and the works of Kuo et al. is that they used a crosslinking agent in the initial stage of the synthesis, while we used it on the second stage of the synthesis. Previously, we also created and investigated cryogels based on gelatin/chitosan/chondroitin sulfate (GelChCS) [35]. In that research we determined the optimal concentration of CS in cryogels. In this work, we aimed to obtain cryogels with biocompatible and chondroinductive properties that could be used in cartilage tissue engineering.

## 2. Results and Discussion

### 2.1. Synthesis and Characterization of GelHACS Cryogels

**Figure 1:**
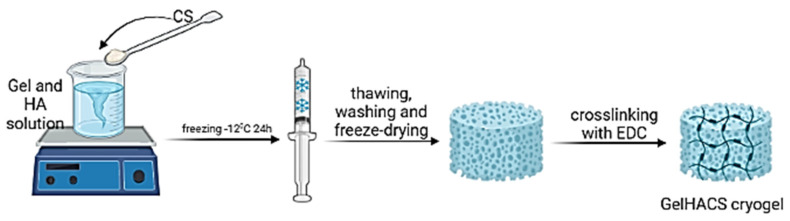
The scheme of a cryogel formation. Created with BioRender. Available online: http://biorender.com (accessed on 11 August 2022).

GelHACS cryogels were synthesized by cryopolymerization method at −12 °C as in our previous study [35]. The ratio between Gel and CS was set to 4:1 by weight and remained unchanged. The formation of cryogels occurred in two stages: in the first phase, physical crosslinking between Gel, HA and CS, and in the second phase, the formation of a chemical bond in cryogels using EDC (Figure 1).

**Figure 2:**
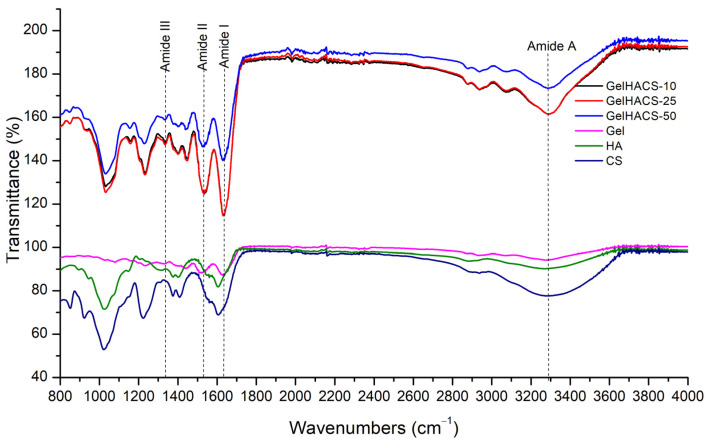
FTIR spectra of GelHACS cryogels.

The presence of the functional groups in the GelHACS cryogels is confirmed by FTIR measurements (Figure 2). The FTIR spectra show a band in the range 3200–3400 cm^–1^, which corresponds to the stretching vibrations of the O–H and N–H functional groups (amide A) involved in the intramolecular hydrogen bond between HA and Gel molecules. Vibrations in the region of 2880 cm^–1^ refer to stretching vibrations of CH- groups. Peaks of 1631, 1541 and 1400 cm^–1^ are responsible to amide I for the C–O stretching vibration, amide II for the NH- bending vibration and the symmetric stretching vibration bands of the carboxyl groups of HA. The absorption band at 1338 cm^–1^ refers to bending vibrations of the amide group (amide III). The absorption peaks at 1233 and 1033 cm^–1^ are assigned to the asymmetric and symmetric stretching vibration bands of the OSO_3_—groups of CS. EDC is used to form covalent bonds between the carboxyl and amine groups of polymers to form amide bonds or ester bonds, resulting in bonds between Gel, HA and CS. No new peaks were observed upon crosslinking with EDC due to peak overlap, which was in a good agreement with the data published previously [2].

### 2.2. Characterization of GelHACS Cryogels

Determination of the gel fraction of the synthesized cryogels showed that with an increase in the content of HA in the cryogel, the content of the gel fraction in the polymer increases from 78.5 ± 1.3 to 88.5 ± 1.2% (Table 1). Apparently, with an increase in the content of HA in cryogels, a faster crosslinking is taking place, leading to an increase in the yield of the gel fraction. It can be noted that cryotropic gelation proceeded efficiently, since all cryogels have a gel fraction yield of more than 75%.

Additionally, with an increase in HA in cryogels, the pore volume (PV) of cryogels increases. Pore volume is an important factor for polymers used in tissue engineering. It is known that polymers with a pore volume above 80% can be used for cell proliferation [50]. Studies have shown that the pore volume of GelHACS25 and GelHACS50 cryogels is at the level of 86%, which was considered beneficial for cell ingrowth and survival. However, the determination of the pore volume of cryogels by the liquid displacement method is not an absolute method and cannot provide complete information about the polymer morphology; therefore, in this work, we carried out an SEM study of the surface of the cryogels.

**Figure 3:**
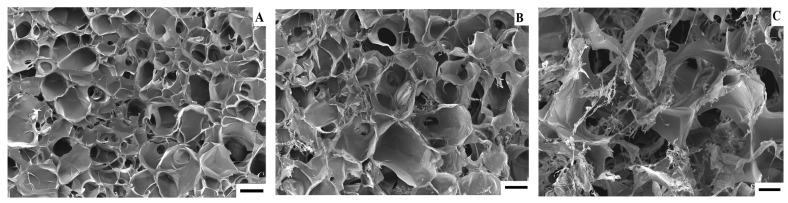
SEM of GelHACS-10 (**A**), GelHACS (**B**) and GelHACS (**C**) cryogels. Scale bar = 100 μm.

Polymers used in tissue engineering must have a porous structure in order to stimulate cell growth to the full extent [3]. For example, according to the SEM results, the pore diameter range is 100–350 μm for GCH and 100–500 μm for GCH/chitosan cryogel. The obtained results showed an excellent result of proliferation of chondrocytes in 21 days [48]. SEM results showed that prepared GelHACS cryogels have open interconnected pore morphology and a macroporous structure (Figure 3 and Figure S1). Mean pore sizes were 84 ± 36, 107 ± 50 and 168 ± 101 µm for GeHACS-10, GelHACS-25 and GelHACS-50, respectively. As can be seen from the results, with an increase in the amount of HA, the surface of the cryogels changes significantly, and filamentous formations characteristic of HA are formed.

**Figure 4:**
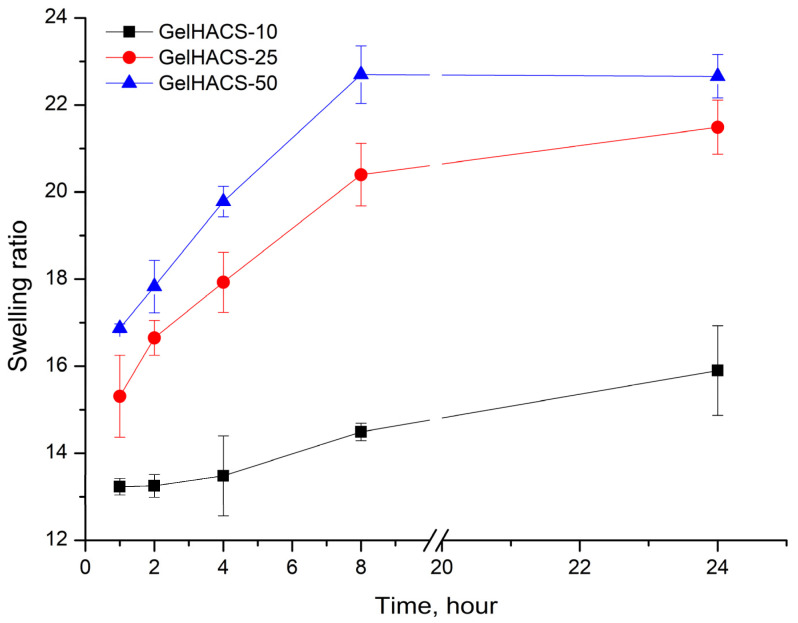
Dependence of the swelling ratio of the cryogels in PBS on the time of swelling.

Changes in the content of HA also affected their osmotic characteristics (swelling extent) of cryogels. The swelling results are shown in Figure 4. The maximum degree of swelling is reached in 24 h. Since cryogels can absorb large amounts of aqueous solutions or liquids and effectively support cell infiltration, this should promote both enzymatic and non-enzymatic degradation [26,51]. Studies have shown that the highest degree of swelling is observed in the GelHACS-50 cryogel (22.7 ± 0.5%), while GelHACS-10 cryogel exhibited the lowest degree of swelling (15.9 ± 1.0%) (Figure 4). As is known, the less the cryogel network is crosslinked, the faster it swells [52]. The SEM results showed that the GelHACS-50 sample has open and large pores with fewer crosslinks, thus it can hold a large amount of liquid in the cryogel spatial network. While GelHACS10 cryogel has smaller pores and a high degree of crosslinking, thereby reducing liquid sorption in the polymer network.

**Figure 5:**
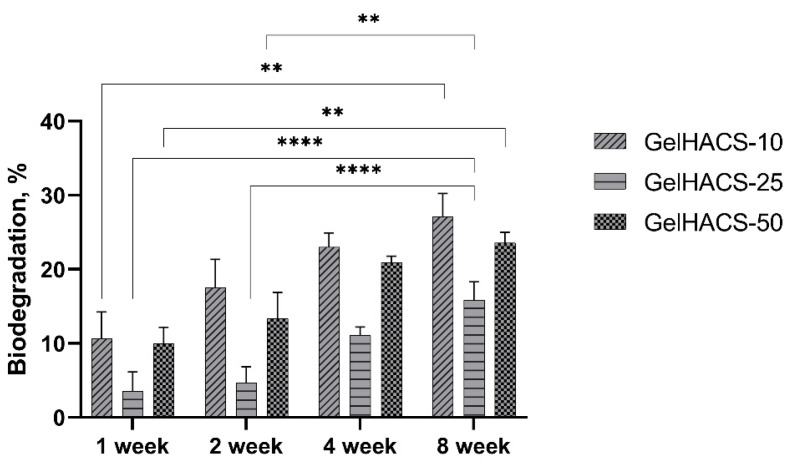
Dependence of the degradation of the cryogels in PBS on the time of degradation. The data are expressed as the means ± SD with ** *p* < 0.01; **** *p* < 0.0001.

The degradation of biomaterials is critical for both drug delivery and tissue engineering, as implanted biomaterial must avoid chronic inflammation, fibrous encapsulation and implant rejection during implantation [26]. During degradation, the process of hydrolysis occurs, which destroys the walls of macropores of cryogels, thereby causing a loss in mass. When studying the degradation of GelHACS-10, GelHACS-25 and GelHACS-50 cryogels in PBS at 37 °C, weight loss was observed over time (Figure 5). In 8 weeks, 27.1 ± 3.1, 15.9 ± 2.4 and 23.6 ± 1.4% of GelHACS-10, GelHACS-25 and GelHACS-50 cryogels, respectively, degraded. The highest degree of degradation was observed for cryogel with the lowest amount of HA, at which Gel degrades to a greater extent due to the crosslinking density and hydrophilic qualities of the polymers. Based on the data obtained, we think that all cryogels have a low degree of degradation and can be further used as scaffolds for cell proliferation.

**Figure 6:**
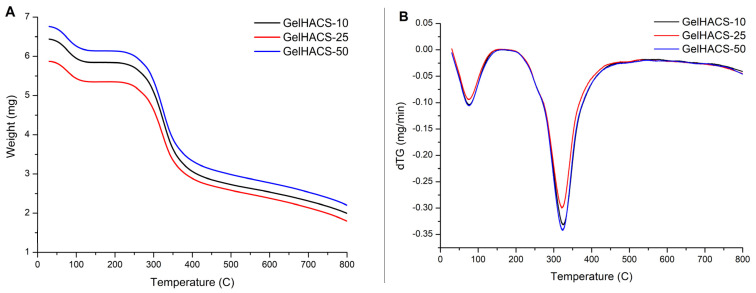

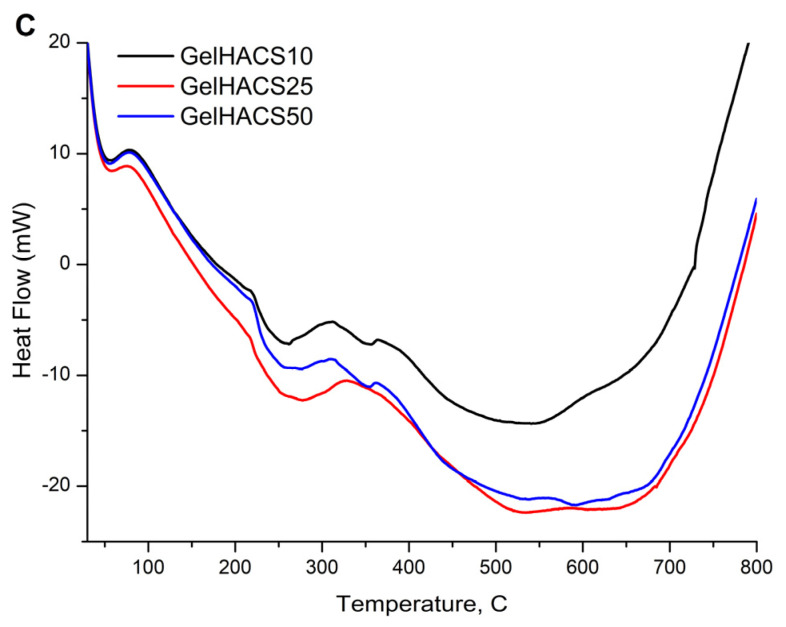
TG (**A**), dTG (**B**) and DSC (**C**) curves of synthesized cryogels.

Thermal properties of cryogels were studied using DSC/dTG/TG curves (Figure 6A–C). The TG and dTG curves (Figure 6A,B) show a weight loss in the range of 100 °C and 300 °C. The first peak is associated with the loss of water in the cryogel matrix, and the second peak corresponds to the destruction and oxidative decomposition of cryogel components. The dTG curves (Figure 6B) also showed similar curves for all samples. At 800 °C, the mass loss was GelHACS-10—69.19%, GelHACS-25—69.51% and GelHACS-50—67.67%. The DSC thermogram (Figure 6C) showed several endo peaks in the region of 50–60 °C, corresponding to the loss of water, then in the region of 250 °C and a broad peak from 350–700 °C, corresponding to the destruction of cryogel components.

### 2.3. Cell Viability Assay and the Proliferation the MSCs on GelHACS Cryogels

**Figure 7:**
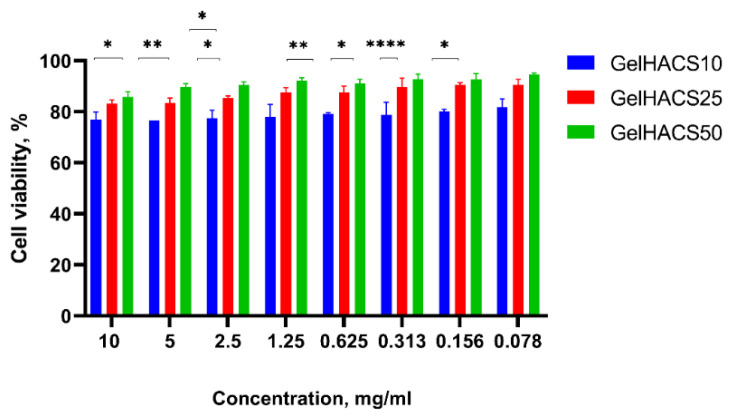
Effects of GelHACS extracts on viability of rat ADMSC presented as percentage of cell viability versus concentration of the extracts. The experiments were repeated in triplicate independently, and the data are expressed as the means ± SD with * *p* < 0.05; ** *p* < 0.01; **** *p* < 0.0001.

The cytotoxic effect of scaffolds towards ADMSC viability was measured by Alamar Blue assay. Rat ADMSCs were incubated at the presence of differing extracts concentrations of scaffolds for 24 h. Figure 7 shows that ADMSCs cultured with extract of GelHACS-50 had the highest viability rates at all concentrations, at the concentration of 10 mg/mL and 0.078 mg/mL the values of viability were 86% and 95%, respectively.

The GelHACS-25 ranked second, showing 81% cell viability at the 10 mg/mL extract’s concentration and 91% at the lowest rate of the dose. Ranked third, GelHACS-10 had the strongest inhibiting effect on cell viability compared to control and other evaluated scaffolds. For instance, 77% of viable cells was detected at the highest dose of GelHACS-10, while the 81.7% cells were detected as viable after 24 h of incubation with 0.078 mg/mL scaffold extract. Taking in account obtained data, all three scaffolds showed lack of cytotoxicity even at the highest concentrations and could be further used as cell scaffolds for regenerative medicine.

### 2.4. Chondrogenic Differentiation of ADMSC Spheroids on Cryogels

**Figure 8:**
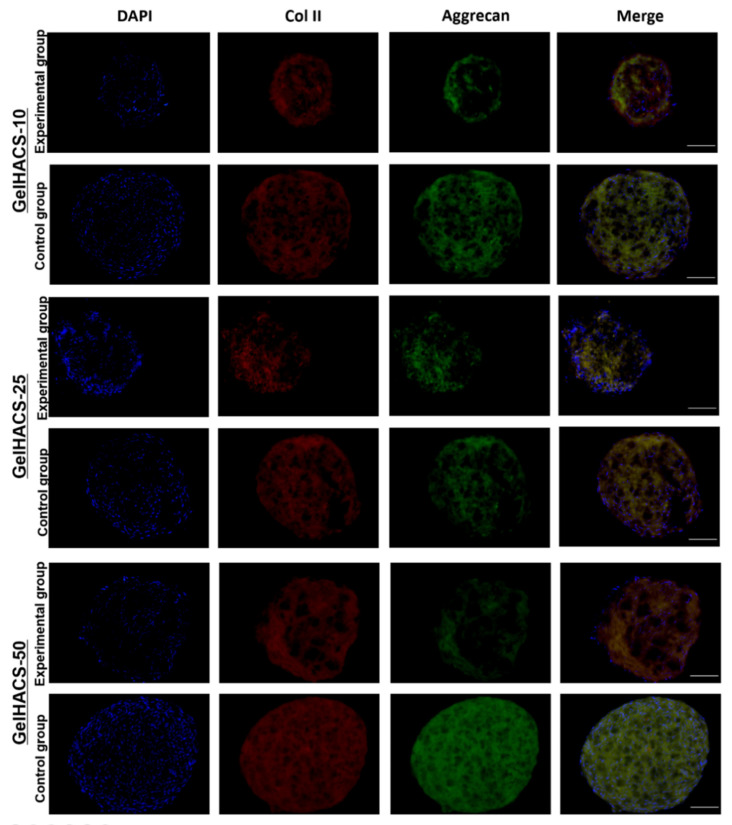
Expression of collagen II and aggrecan in ADMSC spheroids cultured in different cryogels. Red represents collagen II, green aggrecan and blue cell nuclei (blue) are stained by DAPI. Scale bar = 100 µm.

In order to examine the ability of the GelHACS scaffolds to mimic microenvironments specific for hyaline cartilage and induce chondrogenic cell differentiation even in absence of chondrogenic induction agents in vitro, the chondrogenic differentiation assay was performed. As a control groups, the scaffolds with ADMSC spheroids cultivated in culture medium containing chondrogenenic induction agents and TGF-β1 were used. Experimental groups were represented by scaffolds with ADMSC spheroids grown in culture medium without chondrogenenic induction agents and TGF-β1. At the end of the experiment the scaffolds with ADMSC spheroids were immunostained with specific antibodies to aggrecan and collagen type II. Aggrecan and collagen type II were chosen as markers of chondrogenic differentiation due to high abundance of collagen II and aggrecan in cartilage extracellular matrix. Figure 8 shows that expression of both markers were detected in control groups as well as in experimental groups. GelHACS-10, GelHACS-25 and GelHACS-50 showed a strong ability to facilitate chondrogenesis in ADMSC spheroids even in absence of chondrogenic induction agents. The ability of cryogel to mediate cell differentiation alone could extremely facilitate the process of scaffold utilization both as a cell carrier and ready to use bioactive scaffold, which helps enable application of expensive components of induction medium and limits the influence of exterior factors during cultivation.

It is known that the structure, content, pore size and mechanical properties of scaffold have a crucial impact on cell fate and viability as well as on cell proliferation. The sponge structure, which was provided by cryogels, enhances cell–matrix interactions and oxygen and nutrient infiltration. As previously was demonstrated by Zhang and colleagues, human umbilical cord MSCs were able to undergo chondrogenic differentiation after their seeding on decellularized articular cartilage scaffold in the absence of inductive components [53].

Sawatjui et al. showed that gene expression of main chondrogenic markers, such as aggrecan and collagen type II, were significantly increased in human MSCs after their cultivation in constructs comprised of Gel, CS and HA [54]. The analysis of expression of chondrogenic markers such as aggrecan and collagen type II demonstrated that cryogels alone could successfully replicate natural extracellular matrix architecture and could be used as potential scaffolds in biomedicine.

## 3. Conclusions

We have studied the effect of HA on the properties of cryogels based on biopolymers of Gel and CS synthesized by cryopolymerization. It has been established that with an increase in the concentration of HA in the cryogels, the gel content, pore volume, swelling ratio and pore size increase. The MTT assay showed that cryogels do not have toxic properties and can be used as a biological material for cells. All three scaffolds promoted adhesion, growth and chondrogenic phenotype of ADMSC spheroids. The expression of aggrecan and collagen type II in ADMSC spheroids confirm the ability of scaffolds composed of Gel, HA and CS to sufficiently induce chondrogenesis in ADMSC spheroids without any addition of chondrogenic inductive agents as well as accumulate a cartilage-like matrix. Our work demonstrates the potential use of the synthesized cryogels in cartilage tissue engineering and regenerative medicine.

## 4. Materials and Methods

### 4.1. Materials

All of the ingredients were obtained from Sigma-Aldrich and used as received, including gelatin (Type A), hyaluronic acid (sodium salt from Streptococcus equi), *N*-(3-dimethylaminopropyl)-*N*-ethylcarbodiimide hydrochloride (EDC), Dulbecco’s modified Eagle medium (DMEM) and phosphate buffered saline (PBS). Chondroitin sulfate A sodium salt was purchased from Glentham Life Sciences Ltd. (Corsham, UK). All aqueous solutions were prepared using MilliQ water (18.2 MΩ cm).

### 4.2. Preparation of GelHACS Cryogels

A modified technique described previously was adapted for synthesis of cryogels in this work [48]. Using a mechanical rotor Gel (400 mg) and various HA masses (10, 25 and 50 mg) were dissolved in 10 mL of PBS (pH = 7.4) at 37 °C overnight. Gel/HA solution was first treated with CS (100 mg) gradually before being put in syringes and incubated at −12 °C for 24 h. The physically crosslinked polymers resulting from this process were repeatedly rinsed with MilliQ water and PBS (pH = 7.4) after freezing at room temperature. After that, they were frozen at −70 °C and lyophilized. Then, dry cryogels were chemically crosslinked for 16 h using 1% EDC in cold ethanol solution. The resulted cryogels were denoted as GelHACS-10, GelHACS-25 and GelHACS-50 where 10, 25 and 50 are the masses of HA.

### 4.3. Characterization of Cryogels

FT-IR spectra of the samples were recorded using Thermo Scientific Nicolet iS10 FT-IR (Madison, WI, USA) spectrometer. The wavelength range is 4000–400 cm^−1^.

The surfaces of dried cryogels were examined using scanning electron microscope (SEM, Auriga Crossbeam 540, Carl Zeiss, Oberkochen, Germany). The coating of the samples was performed using 10 nm gold. Using ImageJ software, and the average diameter of the pores was calculated.

Thermogravimetry (TG), differential thermogravimetry (DTG) and differential scanning calorimetry (DSC) were used to analyze the thermal behavior of the cryogels using a Simultaneous Thermal Analyzer STA 6000 (PerkinElmer, Waltham, MA, USA) in a nitrogen atmosphere at a heating rate of 10 °C/min in the range of +30 to 800 °C. Gel fraction, swelling, degradation and pore volume of the cryogels were determined using a gravimetric method [35]. The gel fraction was calculated using the masses of cryogels before and after extraction with water (Equation (1)):

(1) $$\mathrm{Gel}\;\left(\%\right)=\frac{W_{w}}{W_{i}}\times 100$$

where W_i_ and W_w_ are the masses of the specimens before and after extraction with water.

The ethanol uptake into the pores was used to estimate the pore volume of the cryogels. The measurements were carried out by immersing dry cryogel specimens containing a mass of W_D_ in absolute ethanol for one day and then recording their final mass W_S_. Pore volume (PV) was calculated by Equation (2):

(2) $$\mathrm{PV}\;\left(\%\right)=\frac{\left(W_{S}-W_{D}\right)}{W_{S}}\times 100$$

The swelling ratio (SR) of the cryogels was calculated by comparing the masses of the swollen sample (W_s_) in 0.1 M PBS (pH 7.4) and the dry sample (W_d_) over time (Equation (3)):

(3) $$\mathrm{SR}=\frac{W_{s}-W_{d}}{W_{d}}$$

Cryogel degradation was measured as follows: cryogels were weighed (W_1_) and transferred to 50 mL tubes filled with sterile 0.1 M PBS (pH 7.4). The tubes were incubated at 37 °C for 4 weeks, with the solution being refreshed twice a week. Cryogel samples were removed from the solution and washed with deionized water at predetermined intervals. After overnight freeze-drying and weighing (W_2_). The degree of degradation (DD) was calculated using the following Equation (4):

(4) $$\mathrm{DD}\left(\%\right)=\frac{W_{1}-W_{2}}{W_{1}}\times 100$$

### 4.4. Isolation of Primary MSC Derived from Rat Adipose Tissue Cultivation and Cell Viability Assessment

All animal procedures were conducted according to the guidelines of the Declaration of Helsinki and approved by the Local Ethics Committee of the National Center for Biotechnology (number NCB-04-2020). The isolation of adipose tissue was performed using Wistar rats (male, 280–330 g) as previously was described [35]. Briefly, after rat sacrifice, adipose tissue was collected and washed in phosphate-buffered saline three times, subsequently chopped into small pieces, and digested in collagenase type I solution (Gibco, Grand Island, NY, USA). Then cell suspension was transferred through a 70 um cell strainer and cultured in Minimum Essential Medium Alpha (Gibco, Paisley, UK) supplemented with 10% fetal bovine serum (Gibco, Paisley, UK) and 1% penicillin/streptomycin with a regular medium change every 2–3 days. The viability of rat ADMSCs in the presence of scaffold was measured by alamarBlue reagent (Invitrogen, Eugene, OR, USA), which contains blue-colored component resazurin serving as an indicator of oxidation-reduction processes in cells. The scaffold extracts were obtained in accordance with ISO 10993-5 and represent the leached eluents of cryogels which are assessed for determine the ability of scaffolds to realize any toxic compounds that could decrease cell viability. Briefly, after 24 h incubation of UV-sterilized GelHACS cryogels in basal alpha-MEM culture media, the eluents were added to cells previously seeded on a 96-well flat-bottom plate in dilutions ranging from 10–0.078 mg/mL in triplicate [55]. At the end of 24 h incubation, medium was replaced by a new medium containing 10% alamarBlue reagent and plates were placed in the incubator for an additional 3–4 h. Afterward, the optical density of processed resazurin was measured using microplate reader (Bio-Rad, Hercules, CA, USA).

### 4.5. Spheroids Formation from Primary Rat ADMSC, In Vitro Cultivation on Cryogels and Immunohistochemistry Analysis

Spheroids were established using the “hanging drops” technique as previously described [56]. On day 3 after cells were seeded in drops, cells formed aggregates which were harvested and placed on the surface of the scaffold in the amount of 20 spheres per cryogel. To evaluate the ability of scaffolds to effectively replicate ECM microenvironment all three scaffolds with spheroids were cultured in medium Dulbecco’s Modified Eagle’s Medium (Gibco, Paisley, UK) supplemented with 10% fetal bovine serum (Gibco, Paisley, UK) and were considered as experimental groups. As control groups scaffolds cultivated in freshly prepared chondrogenic medium on the basis of Dulbecco’s Modified Eagle’s Medium (DMEM, Sigma, Saint Louis, MO, USA) supplemented with 1% ITS, 100 µg/mL sodium pyruvate, 0.1 mM dexamethasone, 50 µg/mL ascorbic acid, 5.33 µg/mL linoleic acid and 10 ng/mL TGF-β1 were used, all medium changes were performed every 2–3 days.

### 4.6. Histological Analysis and Fluorescence Immunohistochemistry

After 14-day cultivation, scaffolds with spheroids were washed twice with PBS and fixed in 4% paraformaldehyde (Sigma, Saint Louis, MO, USA) at 4 °C at room temperature for 30 min followed by washing with PBS, cryopreservation, Tissue-Tek ^®^ OCT compound (Sakura, Gentaur BV, The Netherlands) embedding and freezing at −70 °C. The frozen sections (7 µm thick) were cut using an MNT cryostat (Slee, Nieder-Olm, Germany). After rehydration, scaffolds were washed in TBS containing NaCl (Honeywell, Seelze, Germany), Tris (AppliChem, Darmstadt, Germany) and pH 7.4 supplemented with 0.1% Tween-20 (AppliChem, Darmstadt, Germany). Next, blocking buffer containing 1.5% goat serum (cat#ab7481, Abcam Inc, Toronto, ON, Canada) was added in order to prevent unspecific staining followed by incubation at room temperature for 30 min. Intensive washing procedures were performed and slides were covered with primary antibody dissolved in blocking solution. For detection of chondrogenic differentiation primary antibodies against aggrecan (cat#3773, Abcam Inc, Toronto, ON, Canada) and collagen type II (cat#ab34712, Abcam Inc, Toronto, ON, Canada) were added in 1:200 dilution and incubated overnight at 4 °C, samples with PBS instead of primary antibody were considered as a negative control. After staining with primary antibodies slides were extensively washed and covered with secondary antibodies Alexa Fluor 488 goat anti-mouse (Invitrogen, Eugene, OR, USA) and Alexa Fluor 594 goat anti-rabbit (Invitrogen, Eugene, OR, USA) for 1 h at room temperature followed by washing in TBS. To prevent evaporation, dye fading and to distinguish cell nucleus slides were covered with ProLong™ Gold Antifade Mountant with DAPI (Invitrogen, Eugene, OR, USA). The fluorescence images were taken by Axio Observer A1 (Zeiss, Oberkochen, Germany). All incubations were performed in humidified chamber.

### 4.7. Statistical Analysis

Graphpad Prism (San Diego, CA, USA) and Origin 8.1 (Northampton, MO, USA) statistical software was used to analyze the obtained data. The significant difference between two groups was found using the Student’s *t* test, and the difference between multiple groups was established using the one-way ANOVA (Tukey’s post hoc test). Statistically significant difference was defined as a *p* value 0.05. Data are presented as mean SD.

## Figures and Tables

**Table 1:** The gel fraction and pore volume of cryogels.

Samples	Gel:HA:CS, mg	Gel Fraction (%)	PV (%)
GelHACS-10	400:10:100	78.5 ± 1.3	74.74 ± 1.0
GelHACS-25	400:25:100	84.1 ± 0.8	85.87 ± 0.8
GelHACS-50	400:50:100	88.5 ± 1.2	86.20 ± 0.2

## Data Availability

All data presented in this paper are available upon request from the corresponding author.

## Supplementary Materials

The following supporting information can be downloaded at: https://www.mdpi.com/article/10.3390/gels8090590/s1, Figure S1: SEM images and histogram of pore sizes of the cryogels: (a–c) SEM images of cryogels: GelHACS-10 (a), GelHACS-25 (b), GelHACS-50 (c), scale bar = 100 μm; (d–f) histogram of cryogels: GelHACS-10 (d), GelHACS-25 (e), GelHACS-50 (f).
