# Supplementary material for: Macroporous Cell-Laden Gelatin/Hyaluronic Acid/Chondroitin Sulfate Cryogels for Engineered Tissue Constructs
Source: Gels. 2022 Sep 16;8(9):590. doi: 10.3390/gels8090590 (PMC9498617; doi:10.3390/gels8090590)

Macroporous Cell-Laden Gelatin/Hyaluronic Acid/Chondroitin Sulfate Cryogels for Engineered Tissue Constructs

Gulshakhar Kudaibergen [1,*], Madina Zhunussova [1], Ellina A. Mun [2], Yerlan Ramankulov [1,2] and Vyacheslav Ogay [1]

[1] Stem Cell Laboratory, National Center for Biotechnology, Nur-Sultan 010000, Kazakhstan
[2] School of Science and Humanities, Nazarbayev University, Nur-Sultan 010000, Kazakhstan
* Correspondence: kudaibergen@biocenter.kz

Figure S1. SEM images and histogram of pore sizes of the cryogels: (a-c) SEM images of cryogels: GelHACS-10 (a), GelHACS-25 (b), GelHACS-50 (c), scale bar = 100μm; (d-f) histogram of cryogels: GelHACS-10 (d), GelHACS-25 (e), GelHACS-50 (f).

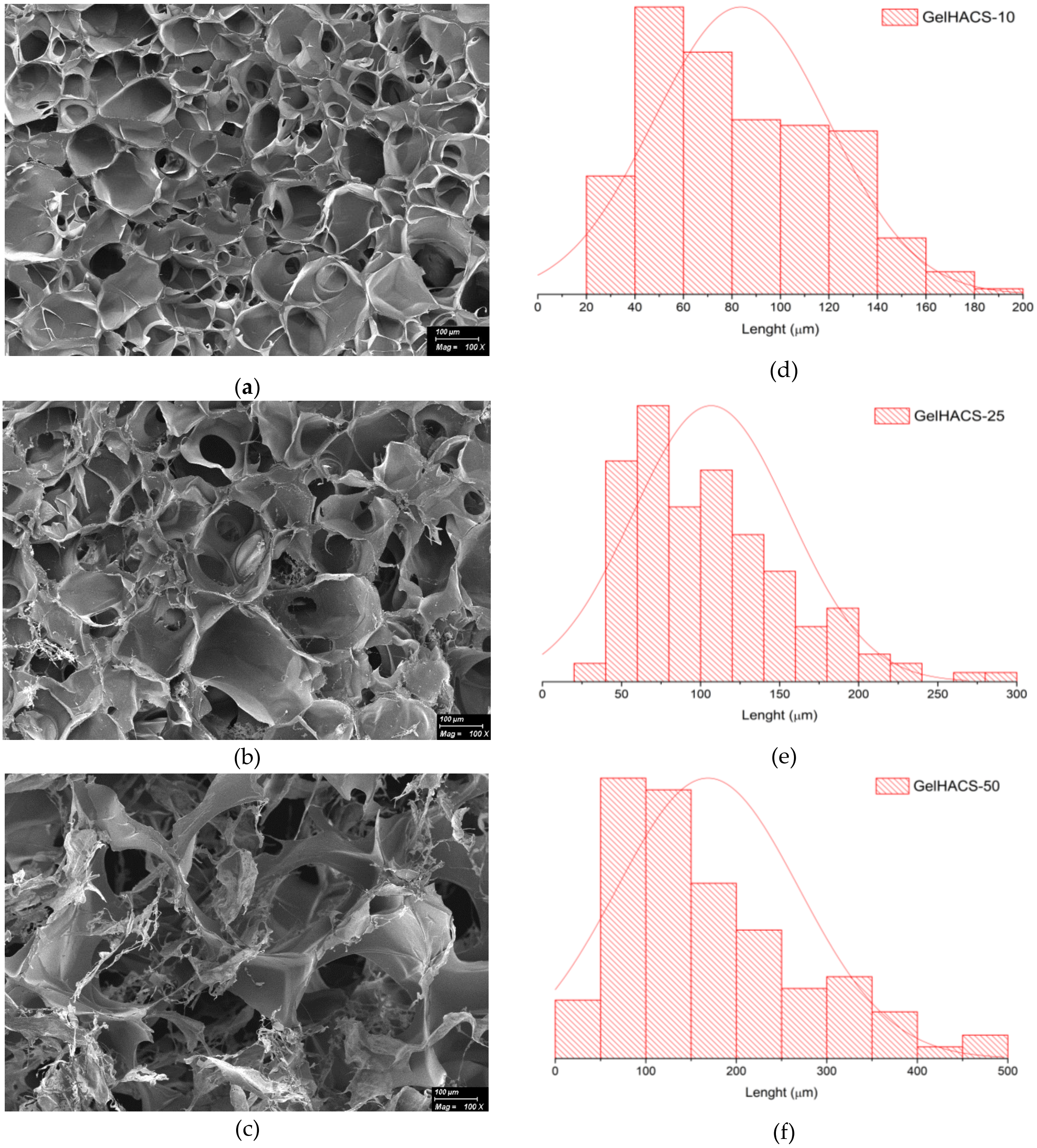

Supplement: Supplementary file 1 [file gels-08-00590-s001.zip › gels-1887896-supplementary.pdf]
